# Tick-borne encephalitis virus (TBEV) – findings on cross reactivity and longevity of TBEV antibodies in animal sera

**DOI:** 10.1186/1746-6148-10-78

**Published:** 2014-04-01

**Authors:** Christine Klaus, Ute Ziegler, Donata Kalthoff, Bernd Hoffmann, Martin Beer

**Affiliations:** 1 Institute of Bacterial Infections and Zoonoses, Friedrich-Loeffler-Institut, Naumburger Str. 96a, D-07743 Jena, Germany; 2 Institute of Novel and Emerging Infectious Diseases, Friedrich-Loeffler-Institut, Südufer 10, D-17493 Greifswald-Insel Riems, Germany; 3 Institute of Diagnostic Virology, Friedrich-Loeffler-Institut, Südufer 10, D-17493 Greifswald-Insel Riems, Germany

**Keywords:** Tick borne encephalitis, Animal sera, Virus neutralization test, ELISA, Cross-reactivity, *Flaviviridae*

## Abstract

**Background:**

By using animal sera as sentinels, natural TBEV foci could be identified and further analyses including investigations of ticks could be initiated. However, antibody response against TBEV-related flaviviruses might adversely affect the readout of such a monitoring. Therefore, the cross-reactivity of the applied TBEV serology test systems – enzyme linked immunosorbent assay (ELISA) and virus neutralization test (VNT) – as well as the longevity of TBEV antibody titres in sheep and goats were investigated in this study.

**Results:**

Cross-reactivity of the TBEV antibody test systems with defined antibody-positive samples against selected members of the *Flaviviridae* family (e.g. Louping ill virus, West Nile virus) was observed for Louping-ill-positive sera only. In contrast, the commercial West Nile virus (WNV) competitive ELISA showed a high level of cross-reactivity with TBEV-specific positive sera.

To assess the longevity of TBEV antibody titres, sera from two sheep and two goats, which had been immunized four times with a commercially available TBEV vaccine, were tested routinely over 28 months. In three of the four animals, TBEV-specific antibody titres could be detected over the whole test period.

In addition, sera from the years 2010 and 2011 were collected in flocks in different villages of Baden-Württemberg and Thuringia to allow re-examination two to four years after the initial analysis. Interestingly, in most cases the results of the former investigations were confirmed, which may be caused by steadily existing natural TBEV foci.

**Conclusion:**

Cross-reactivity must be taken into consideration, particularly for TBEV serology in regions with a prevalence of Louping ill virus and for serological testing of WNV by cross-reactive ELISAs. Furthermore, over-interpretation of single TBEV-positive serological results should be avoided, especially in areas without a TBEV history.

## Background

Tick-borne encephalitis (TBE) is the most important viral tick-borne zoonosis in Europe [1]. TBE virus (TBEV) circulates between ticks and hosts in geographically strictly limited natural foci, which can range in size from large to very small. However, the reason for the patchwork-like spread of TBEV is unclear [2-4]. TBEV prevalence in ticks in these natural foci is very low [5-7], and detection of a natural TBEV focus by collecting and testing ticks is very expensive and time-consuming [8]. Against this background, assessment of the TBEV sero-prevalence in free-ranging animals provides a suitable and valuable additional source of information about TBEV foci in their smaller or larger patchy pattern [9-11].

In general, knowledge on TBE in animals is limited; however infections have been reported in dogs [12-15], monkeys [16,17], and horses [4,18]. Furthermore seroconversion without specific clinical signs of TBE has been described in some animal species such as cattle and small ruminants [11,19,20]. Clinical cases of TBE in ruminants are very seldom; one case of a mouflon (*Ovis ammon musimon*) for example has been reported from Austria [21]. However, these animals are of significant public health relevance. Especially goats and sheep, more rarely cattle, are of importance for the so-called alimentary TBE. During viraemia, the virus is excreted in milk and can be ingested orally by consumption of non-pasteurized milk or cheese produced from raw milk [22-25]. TBE in humans caused by virus-infected milk has occurred in many countries, in the last ten years in Hungary, Austria and Estonia [26-28].

These grazing animals have been shown to be suitable sentinels for the detection of antibodies against TBEV [11], but some questionable points require further examination: (i) the possibility of cross-reactivity of the tested sera with related flaviviruses and (ii) the longevity of TBEV antibody titres in grazing animals.

One example of known cross-reactivity between members of the flavivirus genus comes from serological West Nile virus (WNV) diagnostics [29]. WNV is considered to be the most widespread flavivirus in the world and is transmitted and maintained in an enzootic mosquito-bird cycle [30,31]. Horses can be infected by bridge vectors and develop subclinical infection or neurological disease. False WNV positive ELISA results from horses have been attributed to cross-reactivity with TBEV specific antibodies and complicate monitoring studies on the WNV sero-prevalence [32].

Here we present results on cross-reactivity within the TBEV-specific serology, but also provide a strategy to identify TBEV antibodies as the cause of false positive reactions in serological tests for other flaviviruses using WNV as an example.

## Methods

### Collection of samples

#### Sera positive for Flavivirus specific antibodies

To determine cross-reactivity, five Louping ill virus (LIV) antibody-positive reference sera from sheep (kindly provided by Kim Willoughby, Moredun-Institute, UK) as well as WNV-positive sera from the National Reference Laboratory for WNV (Friedrich-Loeffler-Institut, Greifswald-Insel Riems, Germany) were tested. The WNV sample panel included 15 equine sera (field sera or sera collected after vaccination), and 10 hyperimmune-sera from vaccinated ducks, chickens and rabbits. Additionally, two rabbit sera with antibodies against Usutu virus (USUV) and Japanese encephalitis virus (JEV), respectively, were analyzed.

Furthermore, ascites liquid from mice immunized against TBEV-related viruses such as Russian-spring-summer-encephalitis virus (RSSEV), Powassan virus (POWV), Yellow fever virus (YFV), JEV and St. Louis encephalitis virus (SLEV) could be investigated (kindly provided by Gerhard Dobler, Bundeswehr Institute of Microbiology, Munich, Germany). Each sample was diluted 1:10, 1:50, 1:100, 1:500, and 1:1000 before testing (Table 1).

**Table 1:** Cross-reactivities of TBEV-ELISA and TBEV-VNT results

**Sample number**	**Animal**	**Antibodies classified as (ND**_**50**_**)**	**TBEV-ELISA**	**TBEV-VNT (ND**_**50**_**)**
Serum 1	Sheep	Louping ill-positive	Positive	Positive (1:20)
Serum 2	Sheep	Louping ill-positive	Negative	Negative (1:5)
Serum 3	Sheep	Louping ill-positive	Positive	Positive (>1:40)
Serum 4	Sheep	Louping ill-positive	Positive	Positive (>1:40)
Serum 5	Sheep	Louping ill-positive	Positive	Positive (>1:40)
Serum 6	Horse	WNV positive (1:320)	Negative	Negative (<1:10)
Serum 7	Horse	WNV positive (1:480)	Negative	Negative (<1:10)
Serum 8	Horse	WNV positive (1:1280)	Borderline	Positive (1:40)
Serum 9	Horse	WNV positive (1:160)	Negative	Negative (<1:10)
Serum 10	Horse	WNV positive (1:240)	Negative	Negative (<1:10)
Serum 11	Horse	WNV positive (1:60)	Borderline	Positive (1:80)
Serum 12	Horse	WNV positive (1:40)	Negative	Negative (<1:10)
Serum 13	Horse	WNV positive (1:80)	Negative	Negative (<1:10)
Serum 14	Horse	WNV positive (1:35)	Negative	Negative (<1:10)
Serum 15	Horse	WNV positive (1:120)	Negative	Negative (<1:10)
Serum 16	Horse	WNV positive (1:60)	Negative	Negative (<1:10)
Serum 17	Horse	WNV positive (1:80)	Negative	Negative (<1:10)
Serum 18	Horse	WNV positive (1:240)	Negative	Negative (<1:10)
Serum 19	Horse	WNV positive (1:20)	Negative	Negative (<1:10)
Serum 20	Horse	WNV positive (1:40)	Negative	Negative (<1:10)
Serum 21	Horse	WNV positive (1:60)	Negative	Negative (<1:10)
Serum 22	Horse	WNV positive (1:1280)	Negative	Negative (<1:10)
Serum 23	Chicken	WNV positive (1:500)	Negative	Negative (<1:10)
Serum 24	Chicken	WNV positive (1:250)	Negative	Negative (<1:10)
Serum 25	Chicken	WNV positive (1:80)	Negative	Negative (<1:10)
Serum 26	Chicken	WNV positive (1:900)	Negative	Negative (<1:10)
Serum 27	Duck	WNV positive (1:80)	Negative	Negative (<1:10)
Serum 28	Duck	WNV positive (1:60)	Negative	Negative (<1:10)
Serum 29	Rabbit	WNV positive (1:120)	Negative	Negative (<1:10)
Serum 30	Rabbit	WNV positive (1:480)	Negative	Negative (<1:10)
Serum 31	Rabbit	WNV positive (1:80)	Negative	Negative (<1:10)
Serum 32	Rabbit	WNV positive (1:60)	Negative	Negative (<1:10)
Serum 33	Rabbit	JEV positive (1:60)	Negative	Negative (<1:10)
Serum 34	Rabbit	JEV positive (1:80)	Negative	Negative (<1:10)
Serum 35	Rabbit	USUV positive (1:1280)	Negative	Negative (<1:10)
Serum 36	Rabbit	USUV positive (1:80)	Negative	Negative (<1:10)
Ascites 1	Mouse	RSSEV-positive	Positive*	Positive (1:75)
Ascites 2	Mouse	POWV-positive	Negative*	Negative (<1:25)
Ascites 3	Mouse	YFV-positive	Negative*	Negative (<1:25)
Ascites 4	Mouse	JEV positive	Negative*	Negative (<1:25)
Ascites 5	Mouse	SLEV positive	Negative*	Negative (<1:25)

*all samples diluted 1:10, 1:50, 1:100, 1:500, 1:1000, only results 1:10 are shown in the table, all samples diluted between 1:50 and 1:1000 were negative.

Field sera from horses were collected in two different herds in Bavaria, and sera from TBEV-positive horses [4] were also included (Table 2).

**Table 2:** Cross-reactivity of horse sera between ELISA and VNT

	**TBEV-ELISA**		**TBEV-VNT**	**WNV-ELISA**		**WNV-VNT**
Sample	U/l 1	Result	ND_**50**_	S/N%	Result	ND_**50**_
Serum 1	-2,995	Negative	n.d.	92,25%	Negative	< 1:10
**Serum 2**	**12,500**	**Borderline**	**>1:640/1:960**	**11,99%**	**Positive**	**< 1:10**
Serum 3	-2,938	Negative	n.d.	88,04%	Negative	< 1:10
Serum 4	-3,802	Negative	n.d.	94,11%	Negative	< 1:10
Serum 5	3,859	Negative	n.d.	41,48%	Borderline	< 1:10
**Serum 6**	**17,627**	**Positive**	**1:80**	**7,27%**	**Positive**	**< 1:10**
Serum 7	-2,650	Negative	n.d.	77,31%	Negative	< 1:10
Serum 8	-3,571	Negative	n.d.	86,93%	Negative	< 1:10
Serum 9	-3,168	Negative	n.d.	90,48%	Negative	< 1:10
Serum 10	3,111	Negative	n.d.	3,16%	Negative	< 1:10
Serum 11	-0,806	Negative	n.d.	93,43%	Negative	< 1:10
Serum 12	-3,975	Negative	n.d.	93,54%	Negative	< 1:10
Serum 13	-2,995	Negative	n.d.	92,59%	Negative	< 1:10
Serum 14	-3,744	Negative	n.d.	89,52%	Negative	< 1:10
Serum 15	-2,995	Negative	n.d.	88,38%	Negative	< 1:10
Serum 16	4,896	Negative	n.d.	86,58%	Negative	< 1:10
Serum 17	-3,168	Negative	n.d.	85,80%	Negative	< 1:10
Serum 18	-2,765	Negative	n.d.	91,29%	Negative	< 1:10
**Serum 19**	**17,915**	**Positive**	**1:80**	**15,04%**	**Positive**	**< 1:10**
Serum 20	20,392	Positive	1:240	51,14%	Negative	< 1:10
**Serum 21**	**12,212**	**Borderline**	**1:160**	**13,51%**	**Positive**	**< 1:10**
**Serum 22**	**17,569**	**Positive**	**1:320**	**9,48%**	**Positive**	**< 1:10**
Serum 23	-3,053	Negative	n.d.	84,21%	Negative	< 1:10
Serum 24	-3,399	Negative	n.d.	83,57%	Negative	< 1:10
**Serum 25**	**16,993**	**Positive**	**1:80**	**7,06%**	**Positive**	**< 1:10**
Serum 26	-4,818	Negative	n.d.	89,77%	Negative	< 1:10
Serum 27	-5,867	Negative	n.d.	83,66%	Negative	< 1:10
Serum 28	-3,959	Negative	n.d.	84,43%	Negative	< 1:10
Serum 29	-5,295	Negative	n.d.	91,08%	Negative	< 1:10
Serum 30	10,351	Borderline	<1:5	86,85%	Negative	< 1:10
Serum 31	-5,200	Negative	n.d.	86,23%	Negative	< 1:10
Serum 32	-4,532	Negative	n.d.	86,37%	Negative	< 1:10
**Serum 33**	**31,245**	**Positive**	**>1:40/1:720**	**27,45%**	**Positive**	**< 1:10**
Serum 34	14,168	Positive	>1:40/1:480	76,10%	Negative	< 1:10
**Serum 35**	**21,991**	**Positive**	**1:40**	**40,69%**	**Borderline**	**< 1:10**
**Serum 36**	**35,443**	**Positive**	**>1:40/>1:960**	**21,43%**	**Positive**	**< 1:10**
Serum 37	-5,581	Negative	n.d.	86,84%	Negative	< 1:10
Serum 38	-4,913	Negative	<1:5	88,36%	Negative	< 1:10
**Serum 39**	**35,538**	**Positive**	**>1:40/1:480**	**6,68%**	**Positive**	**< 1:10**
**Serum 40**	**30,291**	**Positive**	**>1:40/1:960**	**17,62%**	**Positive**	**< 1:10**
Serum 41	-5,200	Negative	<1:5	85,33%	Negative	< 1:10
Serum 42	-3,864	Negative	n.d.	86,76%	Negative	< 1:10
Serum 43	-5,486	Negative	n.d.	87,18%	Negative	< 1:10
Serum 44	-3,959	Negative	n.d.	87,91%	Negative	< 1:10
Serum 45	-5,200	Negative	n.d.	86,37%	Negative	< 1:10
**Serum 46**	**13,691**	**Positive**	**>1:40/>1:960**	**3,07%**	**Positive**	**< 1:10**

In bold = TBEV-positive horses would result in false positive outcomes in the commercial WNV-ELISA, but clearly negative in the WNV-specific VNT.

n.d. = not done.

#### Sera collected to analyze the longevity of the humoral immune response

Two goats and two sheep were immunized four times as described by Klaus et al. [33] and TBEV antibody titres were traced over a period of 28 months (Table 3).

**Table 3:** Longevity of TBEV-antibody titres (VNT) in two immunized goats and sheep

Month*	Goat 1**	Goat 2**	Sheep 1**	Sheep 2**
0	<1:5	<1:5	<1:5	<1:5
6	1:60	1:80	1:80	1:80
12	1:80	1:120	1:80	1:80
18	1:60	1:320	1:10	1:20
24	1:30	1:120	1:30	1:20
28	1:80	1:80	1:30	1:60

*sera examined every month, only selected data shown.

**immunized in week 0, 1, 3 and 11 with one dose of FSME-IMMUN Erwachsene (Baxter Deutschland GmbH, Germany), as described in [33].

<1:5 = negative.

In addition, different field sera were included in the analysis: sera from sheep and goat flocks from the TBE risk areas “Ortenaukreis”, “Bodenseekreis” and “Breisgau-Hochschwarzwald (HS)”, and sera from the TBE non-risk area “Altenburger Land” which were collected in 2010 and 2011 (Table 4). Although the sera did not come from the same individual animals, the datasets were compared to the sero-prevalence results exhibited by the same flocks in 2006 and 2009 [11].

**Table 4:** Re-tested goat flocks for TBEV-specific antibodies (Thuringia-TH, Baden-Wuerttemberg-BW) and sheep flocks (Baden-Wuerttemberg-BW), re-collected in 2010/11

**Country**	**District**	**Flock (village)**	**Year**	**Species**	**No. of sera**	**No. of positive sera**	**Seroprev. (%)**
BW	Bodenseekreis	S1 (Salem)	2011	sheep	100	13	13
BW	Bodenseekreis	S1 (Salem)	2008-09*	sheep	100	9	9
BW	Breisgau (HS)	Sulzburg	2010	goats	28	5	17,8
BW	Breisgau (HS)	Sulzburg	2006-09*	goats	28	12	43
BW	Breisgau (HS)	different flocks**	2010	goats	355	4	1,1
BW	Breisgau (HS)	different flocks**	2006-09*	goats	349	13	3,7
BW	Ortenaukreis	Nordrach	2010	goats	28	12	42,8
BW	Ortenaukreis	Nordrach	2008*	goats	20	12	60
BW	Ortenaukreis	various flocks***	2010	goats	100	15	15
BW	Ortenaukreis	various flocks	2008*	goats	355	51	14,4
TH	Altenburger Land	A1 (Altkirchen)	2011	goats	90	0	0
TH	Altenburger Land	A1 (Altkirchen)	2009*	goats	73	5	7

*in detail published in [11].

**re-tested flocks from Horben, Münstertal and Löffingen-Dittishausen, results not shown in detail.

***flocks from Haslach, Kürzell-Meißenheim, Offenburg, Hohberg, results not shown in detail.

In addition, in Salem, district Bodenseekreis, not only sera but also ticks were collected in 2011 (89 female, 116 male, 1537 nymphs, 46 larvae) in the vicinity of the monkey mountain, where in 2006 TBEV strain Salem had first been isolated from a monkey (*Macaca sylvanus*) [16,17] and sheep sera had been collected and examined in 2008/2009 [34], but TBEV-RNA in ticks has not been detected so far. Ticks were pooled (up to 5 female, 5 male, 10 nymphs, 20 larvae), examined for TBEV-RNA and positive pools were sequenced as described [4].

### Test systems

#### TBEV specific serology

All sera were examined by means of a two-step method [33,34], which consisted of (I) first screening with a modified TBEV-antibody ELISA (Immunozym FSME IgM kit, Progen GmbH, Heidelberg, Germany) allowing analysis of the whole immunoglobulin fraction and (II) subsequent confirmation of all ELISA-positive or questionable results by a virus neutralization test (VNT; [10]). Only sera confirmed by VNT were finally classified as TBEV antibody positive.

#### WNV specific serology

For detection of WNV-specific antibodies, horse samples were investigated by a commercially available competition ELISA, which allows the species-independent recognition of WNV antibodies against the PrM- and E envelope protein (ID Screen® West Nile Competition, IDVet, Grabels, France). Additionally, all serum samples were tested for neutralizing WNV antibodies by VNT with the WNV strain NY 99 (lineage 1, accession no. AF196835) and/or strain Austria (lineage 2, accession no. HM015884, kindly provided by Dr. N. Nowotny, Institute of Virology, University of Veterinary Medicine, Vienna) as described previously [35].

## Results

### Cross-reactivity in TBEV-serology

The majority of samples from the tested Flavivirus-antibody-positive-panel (Table 1) showed clearly negative results using the TBEV-ELISA. Sera with specific antibodies to non-TBEV flaviviruses*,* such as WNV, JEV, USUV, YFV, SLEV or POWV, scored also clearly negative using the TBEV-ELISA. Nevertheless, four out of five LIV-antibody-positive sheep sera reacted positive in the TBEV-ELISA as well as in the TBEV-VNT. In addition, RSSEV reactive mouse ascites scored also positive in both, the TBEV-ELISA and the TBEV-VNT, but only at the lowest dilution of 1:10 (Table 1).

### Cross-reactivity in WNV serology

The fifteen WNV-positive horse sera were classified as clearly negative in the TBEV-ELISA, which was confirmed by the TBEV-VNT. Furthermore, 10 WNV-specific hyperimmune sera from vaccinated chickens, ducks and rabbits were clearly negative both in the TBEV-ELISA and the TBEV-VNT. The same could be shown for the two hyperimmune sera from JEV and USUV vaccinated rabbits, respectively (Table 1). Therefore, WNV-specific antibodies did not obviously cross- react with our TBEV-specific serology testing.

In contrast, WNV-negative (verified by WNV-specific VNT) horse sera scored positive in the commercial WNV-ELISA. Here it could be verified that TBEV-specific antibodies (ELISA and VNT) were the source of cross-reactivity. These results therefore indicate that TBEV-specific antibodies did interfere with WNV-specific ELISA diagnostics and have to be taken into account (Table 2).

### Longevity of TBEV-specific antibody titres in immunized animals and re-testing of sentinel flocks for long-term observations

The development of TBEV-specific antibody titres over a time span of more than two years is shown in Table 3. TBEV-specific antibody titres increased in the vaccinated sheep and goats until 18 weeks after the first immunization and started to decrease to lower VNT titres over the following weeks to reach lower but still positive titres 28 months after vaccination (Table 3).

In order to track the development of TBEV-antibodies in the field situation, selected flocks in Baden-Wuerttemberg and Thuringia (Table 4) were repeatedly tested within a time interval of one to four years. Interestingly, the initial results reported for the districts “Bodenseekreis”, “Breisgau-HS”, and “Ortenaukreis” (Baden-Wuerttemberg), were confirmed [11]. For example, in the sheep flock in Salem (district “Bodenseekreis”) a TBEV-sero-prevalence of 13% was detected (in 2008/2009: 9%), in the goat flock in “Sulzburg” (district “Breisgau-HS”) 17.8% were seen (2006–09: 43%), and in a goat flock in “Nordrach” (district “Ortenaukreis”) 42.8% scored positive (2008: 60%). These findings are in good agreement with the TBE-risk-area status as determined by the Robert Koch-Institute [36,37].

All animal experiments in this study were conducted in strict accordance with a high standard of veterinary care. The protocols were approved by the competent authority of the Federal State of Thuringia, Thuringian State Office for Food Safety and Consumer Protection, Dept. of Health Protection, Veterinary Medicine and Pharmacy, Bad Langensalza, Thuringia, Germany (Reg. no. 04-104/10) and reviewed and approved by the competent authority of the Federal State of Mecklenburg-Western Pomerania, State Office for Agriculture, Food Safety and Fisheries, Rostock, Mecklenburg-Western Pomerania, Germany (LALLF M-V/TSD/7221.3-2.5.1-002/11).

In addition to these serological results in Salem (Bodenseekreis) 1788 collected ticks in 208 pools were analyzed by TBEV-specific RT-qPCR and in one pool of 10 nymphs collected around the monkey mountain TBEV-RNA was detected. The nucleotide sequence (2866 nt in total) of the structure protein genes of TBEV were determined (GenBank KC292217) and compared with the TBEV sequences available in the NCBI GenBank database using BLAST. The highest homology with 99.55% was ascertained for TBEV strain Salem (GenBank FJ572210). Here 2853 nt of the compared 2866 nt were identical.

**Figure 1:**
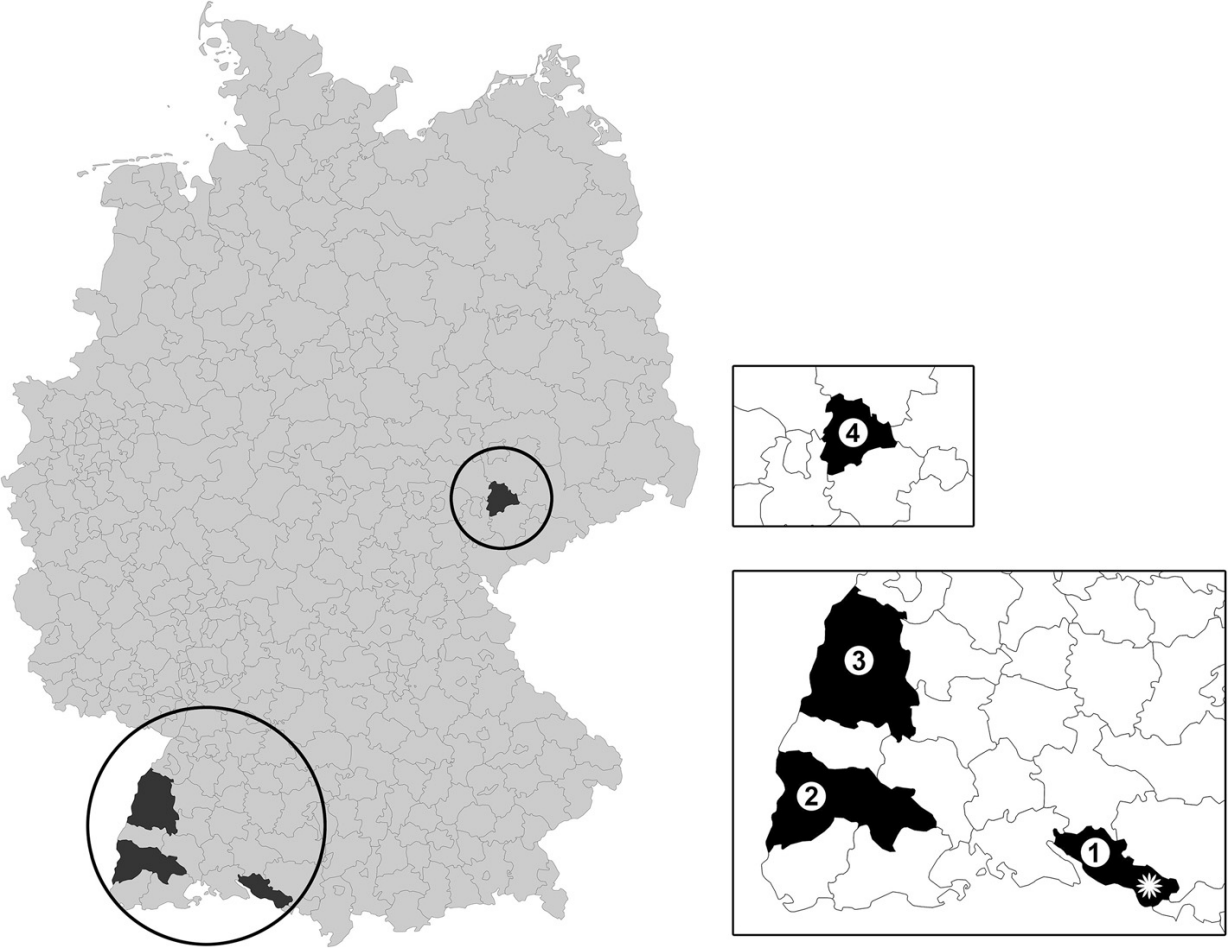
**Places of re-tested flocks in Baden-Wuerttemberg and Thuringia.** 1: Bodenseekreis; 2: Breisgau-Hochschwarzwald; 3: Ortenaukreis; 4: Altenburger Land; star: in addition tick collection site.

Interestingly, the results from a flock in Thuringia surveyed in 2009 could not be confirmed: In 2009, surprisingly five out of 73 sera in this goat flock in “Altkirchen” in the district “Altenburger Land”, Thuringia, which is defined as a TBE non-risk area, tested TBEV-antibody-positive [11]. In 2011, we tested 90 sera from this flock and did not find any TBEV-antibody-positive sera (Table 4). The map (Figure 1) shows the four districts where sera were collected.

## Discussion

A limited cross-reactivity of non-TBEV, but flavivirus-antibody-positive sera was observed in the TBEV-specific test strategy (ELISA and VNT) used for the selection of regions with a risk for TBEV infection. Four of five sera defined as LIV-antibody positive and ascites from a RSSEV-infected mouse reacted positive in the ELISA as well as in the VNT assay (Table 1). Therefore, TBEV-serology in most cases is not able to differentiate between LIV-antibody-positive, RSSEV-antibody-positive and TBEV-antibody-positive sera, and an intense cross-reactivity of sera specific for these related viruses must be assumed. Phylogenetic analysis of the genus Flavivirus confirmed the close relationship of LIV, RSSEV and TBEV [38]. However, since to our knowledge human TBE cases have not been described in Great Britain [1,39], it must be concluded that double infection with Louping ill and TBEV as a reason for these results can be excluded. Cross-reactivity would only be relevant in countries where both diseases are present. In Germany, Louping ill as well as RSSE has not been observed to date [39]. In addition, clinical signs of Louping ill in sheep are severe, while TBE infection in ruminants normally is subclinical [39-41].

Furthermore, no cross-reactivity was observed between our TBEV test system (ELISA and VNT) and defined WNV-positive horse sera (Table 1).

It is well-known, that the used commercial competitive WNV-ELISA cross-reacts with other flaviviruses [29,32], necessitating the use of virus-specific neutralization assays for the final determination of the etiologic flavivirus. Here, the cumulative occurrence of false-positive WNV-results with the used commercial WNV-ELISA system (Table 2) is the consequence of a clear cross-reactivity with TBEV-specific antibodies. In this case, a reasonable discrimination is only possible by means of a virus-specific neutralization assay. The results of this study are therefore in good agreement with those of a previous study [29]. In Germany, TBEV-related diseases in horses have been reported recently [4,42]. Müller et al. [42] investigated a population of 240 horses in the endemic region of Marburg-Biedenkopf and identified 2.9% horses with TBEV-neutralizing antibodies. Consequently, TBEV should be considered as an important and relevant differential diagnosis for WNV infections in horses, although there is no evidence for indigenous WNV infections in Germany so far [32]. Our results are in accordance with a similar study in Austria, where an unexpectedly high seroconversion rate against TBEV was identified in a large herd of horses [43]. Therefore, our TBEV test system could also be helpful for clarifying non-specific serological WNV antibody-reactive results in regions without a WNV history.

On the other hand, testing of horses can even be used as an indicator to detect TBEV-affected areas and thus identify a TBEV focus.

In summary, our TBEV antibody test system is well suited and, despite a partial cross-reactivity to LIV- and RSSEV-antibodies, cross-reactivity with other flaviviruses is not to be expected in countries like Germany. However, the number of samples tested so far has been low, so these findings should be verified in future studies.

In our investigations on the longevity of TBEV antibody titres, only small differences were seen between the two immunized goats and sheep. TBEV-specific antibody titres were still detectable after 28 months with lower but specific titres in the two goats and two sheep (Table 3). While the progress of TBEV antibody titres reflects the situation after immunization with an inactivated vaccine, natural infection might result in relatively similar humoral immune responses; e.g. in horses it was possible to re-test naturally infected TBEV-antibody-positive animals. In two flocks in Bavaria, all six TBEV antibody-positive horses which were re-tested after nine months had VNT-positive titres and it was also possible to detect TBEV in ticks collected in the vicinity of the stables [4]. Also Leschnik et al. [14] observed TBEV titres in dog sera for nine months after natural infection.

In addition, in repeatedly tested goat and sheep flocks the detected sero-prevalences were in good accordance with the epidemiological TBEV situation in the surroundings. In the district “Bodenseekreis”, a defined TBE risk area with a very long TBEV history, 60 human cases were registered between 2001 and 2012, and the existence of a TBEV natural focus in Salem described in animals in 2006 [16,17,34] was re-confirmed by TBEV antibody-positive sheep sera (Table 4). Detection of TBEV-RNA (GenBank KC292217) closely related to the TBEV strain “Salem“(GenBank FJ572210) in one pool of 10 nymphs among 1788 collected ticks also confirmed the serological results (data not shown in detail). This strain was first isolated and sequenced from a monkey (*Macaca sylvanus*) from the monkey mountain Salem with severe neurological clinical symptoms in 2006 [16,17]. However, until our here presented results TBEV-RNA could not be found in ticks at this place. . After our investigations in horse herds in Bavaria [4] it was an additional case where sera from grazing animals as sentinels were successfully used for a targeted tick collection to detect TBEV-RNA.

The districts “Breisgau-HS” and “Ortenaukreis” also have a well-known TBEV history with 64 (Breisgau-HS) and 246 (Ortenaukreis) human cases, respectively, between 2001 and 2012.

In the goat flock in “Altkirchen”, district “Altenburger Land” (Thuringia), however, re-testing provided a different unexpected result. Surprisingly, in 2009, five of 73 sera from this TBE non-risk area with so far no human TBE cases had been found to be TBEV antibody-positive (7%). In 2011, 90 sera from new goats of this flock were re-tested and no TBEV antibody-positive results were detected (Table 4). One reasonable explanation might be the elimination of the relevant animals from the flock, since persistence of TBEV-specific antibody titres is possible for more than 28 months.

Therefore, detection of a small number of TBEV antibody-positive sera in TBEV non-risk areas with no human TBE cases should be interpreted with care (animals might originate from TBE risk areas). However, the longevity data support the conclusion that sera from grazing animals are well suited as a valuable source of serological data supporting the TBEV status of an area.

## Conclusions

TBEV-specific sero-prevalences in animals are an additional valuable tool that can help identify natural TBEV foci which are in most cases spread in a patchwork-like pattern. Cross-reactivity is rare and does not play a significant role in countries without a prevalence of a broad range of TBEV-related flaviviruses. Furthermore, TBEV is a very important and relevant cause for false-positive WNV-ELISA results in horses and must be taken into consideration in monitoring or sentinel programs.

Finally, all TBEV serology results should be interpreted in close cooperation between human and veterinary medicine, and recommendations for the vaccination of humans, based exclusively on the TBEV sero-prevalence in animals should be avoided.

## Competing interests

The authors declare that they have no competing interests nor did they have any competing financial interests in relation to the work described.

## Authors’ contributions

CK collected sera from immunized animals, carried out ELISA, evaluated and interpreted data of TBEV, wrote the manuscript; UZ evaluated and interpreted data of WNV, wrote this part of manuscript; DK developed and carried out VNT; BH developed and carried out RT-qPCR; MB designed the study and drafted the manuscript. All authors revised the manuscript critically and approved the final version.
